# Genome-Wide Identification and Analysis of WD40 Family and Its Expression in *F. vesca* at Different Coloring Stages

**DOI:** 10.3390/ijms252212334

**Published:** 2024-11-17

**Authors:** Hongyu Yang, Wenxia Yao, Xiangjun Fan, Yang Lu, Yan Wang, Zonghuan Ma

**Affiliations:** College of Horticulture, Gansu Agricultural University, Lanzhou 730070, China

**Keywords:** *FvWD40* gene family, bioinformatics analysis, expression analysis

## Abstract

WD40 proteins play important roles in the synthesis and regulation of anthocyanin, the regulation of plant morphology and development, and the response to various abiotic stresses. However, the role of WD40 in *Fragaria vesca* (*F. vesca*) has not been studied. In this study, a total of 216 FvWD40 family members were identified, which were divided into four subfamilies based on evolutionary tree analysis. Subcellular localization predictions show that FvWD40 family members are mainly localized in chloroplasts, nuclei, and cytoplasm. An analysis of collinearity revealed a total of eight pairs of intraspecific collinearity of the *FvWD40* gene family, and interspecific collinearity showed that the *FvWD40* gene family covaried more gene pairs with *Arabidopsis thaliana* (*Arabidopsis*) than with rice (*Oryza sativa*). Promoter cis-acting elements revealed that the *FvWD40* gene family contains predominantly light, hormone, and abiotic stress response elements. Tissue-specific expression analysis showed that a number of members including *FvWD40-111* and *FvWD40-137* were highly expressed in all tissues, and a number or members including *FvWD40-97* and *FvWD40-102* were lowly expressed in all tissues. The *FvWD40* gene family was found to be expressed at all four different coloring stages of *F. vesca* by qRT-PCR, with lower expression at the 50% coloring stage (S3). *FvWD40-24*, *FvWD40-50*, and *FvWD40-60* showed the highest expression during the white fruit stage (S1) period, suggesting that these genes play a potential regulatory role in the pre-fruit coloring stage. *FvWD40-62*, *FvWD40-88* and *FvWD40-103* had the highest expression at the 20% coloration stage (S2), and *FvWD40-115*, *FvWD40-170*, *FvWD40-184* and *FvWD40-195* had the highest expression at the full coloration stage (S4). These results suggest a potential role for these genes during fruit coloration. This study lays a foundation for further research on the function of the *WD40* gene family.

## 1. Introduction

WD40 protein, also known as WD40 repeats, is widespread in all eukaryotes and relatively rare in prokaryotes [1]. The WD40 motif in WD40 proteins is highly conserved, usually containing about 40–60 amino acid residues, with a glycine–histidine (GH) dipeptide at the N-terminus and a tryptophan-aspartic acid (WD) dipeptide at the C-terminus [2,3,4]. WD40 is a relatively large gene family in plants and has been identified in a variety of plants, such as in *Arabidopsis* [5], eggplant [6], tomato [7], and sugar beet [8]. The presence of WD40 protein has been reported in only a small percentage of archaea (27/134) and bacteria (466/1679) [9]. Comparatively speaking, the identification and function of *WD40* genes have been more widely studied in animals and plants. WD40 repeat proteins are involved in complex biological regulatory mechanisms through interactions with a variety of proteins. These mechanisms range from the control of gene expression, such as DNA replication and processing in response to damage, to the regulation of chromatin structure, i.e., histone recognition. In addition, WD40 proteins are involved in the fine tuning of gene transcription, further processing of protein function, i.e., post-translational modification, and signaling processes inside and outside the cell. They are also involved in protein degradation pathways and the regulation of apoptosis [10,11,12]. WD40 proteins play multiple roles in plants and their main functions can be summarized as follows: influencing plant growth and developmental processes [13,14], regulating anthocyanin synthesis [15], and enhancing plant adaptation to biotic and abiotic environmental stresses [16]. It has been shown that the WD40-related protein NtTTG1 enhances drought tolerance in tobacco [17]. WD40 protein also plays an active role in high temperature and NaCl stress [18]. Zhang et al. found that the protein Ehd5, which contains the WD40 structural domain, regulates flowering in rice [19].

Anthocyanins are widely found in plants and are important secondary metabolites of plants. The accumulation of anthocyanins can help plants resist adversity and pathogen invasion, and can attract insects for pollination [20,21]. The genes affecting anthocyanin synthesis are mainly categorized into structural genes and transcription factors. Structural genes include *phenylalanine aminolytic enzyme* (*PAL*), *chalcone synthase* (*CHS*), *chalcone isomerase* (*CHI*), *flavonoid hydroxylase* (*F3H*), *flavonoid 3′-hydroxylase* (*F3′H*), *dihydroflavonoid reductase* (*DFR*), *anthocyanidin synthesis* (*ANS*), and *flavonoid glycosyltransferases* (*UFGT*) [22]. Transcription factors mainly include *MYB*, *bHLH* and *WD40*. Studies have shown that the MYB-bHLH-WD40 (MBW) transcriptional complex is widely present in many plants and regulates the synthesis of anthocyanins in plants, and that environmental factors can affect anthocyanin accumulation by influencing the MBW transcriptional complex [23,24,25,26].

*AN11* in *Petunia hybrida* was the first reported *WD40* gene related to anthocyanin synthesis, and it regulates the expression of genes related to anthocyanin synthesis mainly through post-translational modification of the downstream MYB transcription factor AN2 [27]. The *AtTTG1* gene in *Arabidopsis* and the *PAC1* gene in maize (*Zea mays*) were subsequently reported to contain WD40 repeat proteins, which function similarly to the anthocyanin synthesis-related gene *PhAN11* in regulating the synthesis of anthocyanins [28,29]. An et al. [30] cloned the WD40 protein gene *MdTTG1* in apple, which was identified as a gene related to anthocyanin accumulation by expression analysis, and found that it interacted with bHLH transcription factor to enhance the accumulation of anthocyanin in mustard. By knocking out the *MtWD40-1* gene in alfalfa, a significant reduction in the content of proanthocyanidins and anthocyanidins was observed in the mutant plants, which suggests that the *MtWD40-1* gene plays a role in promoting anthocyanin accumulation in alfalfa [31]. This indicates that the gene positively regulates the accumulation of anthocyanin. In figs (*Ficus carica* L.), FcWD40-97 interacts with FcMYB114, FcMYB123, and FcbHLH42 to form an MBW complex that co-regulates anthocyanin synthesis [32]. In *Raphanus sativus*, heterologous expression of the *WD40* gene *RsTTG1* in an *Arabidopsis* mutant restored anthocyanin and PA accumulation in the mutant, and *RsTTG1* stabilized the activation of the promoters of the anthocyanidin-synthesizing genes *RsCHS* and *RsDFR* when the RsTTG1 protein was coexpressed with the RsTT8 protein [33]. The eggplant WD40-56 protein may play a key role in determining the color formation of aubergine fruits by regulating structural genes associated with anthocyanin synthesis [6]. In blue sterile flower varieties of hydrangea, the *HmWDR68* gene interacts significantly with several key genes in the anthocyanin synthesis pathway, including *HmF3H*, *HmC3′5′H*, *HmDFR*, and *HmANS* [34].

*WD40* is an important gene that regulates anthocyanin synthesis and is important for fruit coloration. The MBW complex has been reported on the regulation of fruit coloration in pineapple strawberries, but it has not been reported in the WD40 single gene in forest strawberry coloration [35,36]. Based on this study, we identified the members of the *F. vesca WD40* gene family from the *F. vesca* genome, performed bioinformatics analysis, and analyzed the changes in the expression levels of some of the members in four periods by real-time fluorescence quantitative PCR, which clarified the roles of some of the members in regulating anthocyanoside synthesis in fruits, and provided candidate genes for the in-depth study of their functions in the later stage of this study.

## 2. Results and Analysis

### 2.1. Identification, Physicochemical Properties, and Secondary Structure Analysis of F. vesca WD40 Gene Family

**Figure 1:**
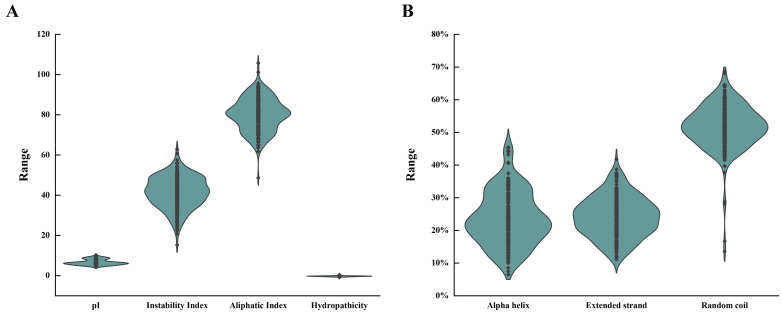
Physicochemical properties and secondary structure analysis of the *FvWD40* gene family. (**A**) Analysis of physicochemical properties of the *FvWD40* gene family. (**B**) Secondary structure analysis of the *FvWD40* gene family.

In this study, a total of 216 *WD40* genes were identified in strawberries, which were named *FvWD40-1-FvWD40-216* according to their location on the chromosome. Based on the physicochemical property analysis, the isoelectric point of the *FvWD40* gene family was shown to be between 4.36 and 10.28. The instability index ranged from 15.27 to 62.94, the aliphatic index from 48.65 to 105.73, and the hydrophilicity from −0.89 to 0.45 (Figure 1A). Secondary structure prediction revealed that all *FvWD40* genes were devoid of β-turns and consisted mainly of α-helix, random coil, and extended strand. The α-helix ranged from 6.47% to 45.44%, the extended strand from 11.13% to 41.81%, and the irregular coils from 13.58% to 68.67% (Figure 1B).

### 2.2. Evolutionary Tree, Motif, and Gene Structure Analysis of F. vesca WD40 Gene Family

**Figure 2:**
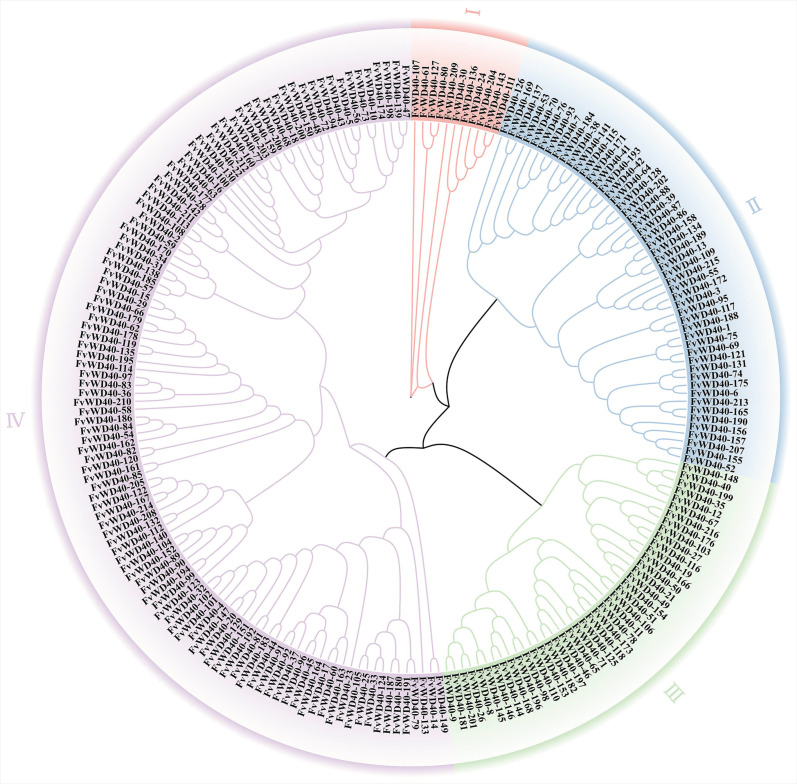
Phylogenetic developmental analysis of FvWD40 family proteins. Roman numerals represent different subgroups.

The 216 FvWD40 proteins were subjected to evolutionary tree analysis and classified into four subfamilies. Subfamily I had the fewest family members and subfamily IV had the most family members. Subgroups I–IV contained 11, 51, 42, and 112 members, respectively (Figure 2).

**Figure 3:**
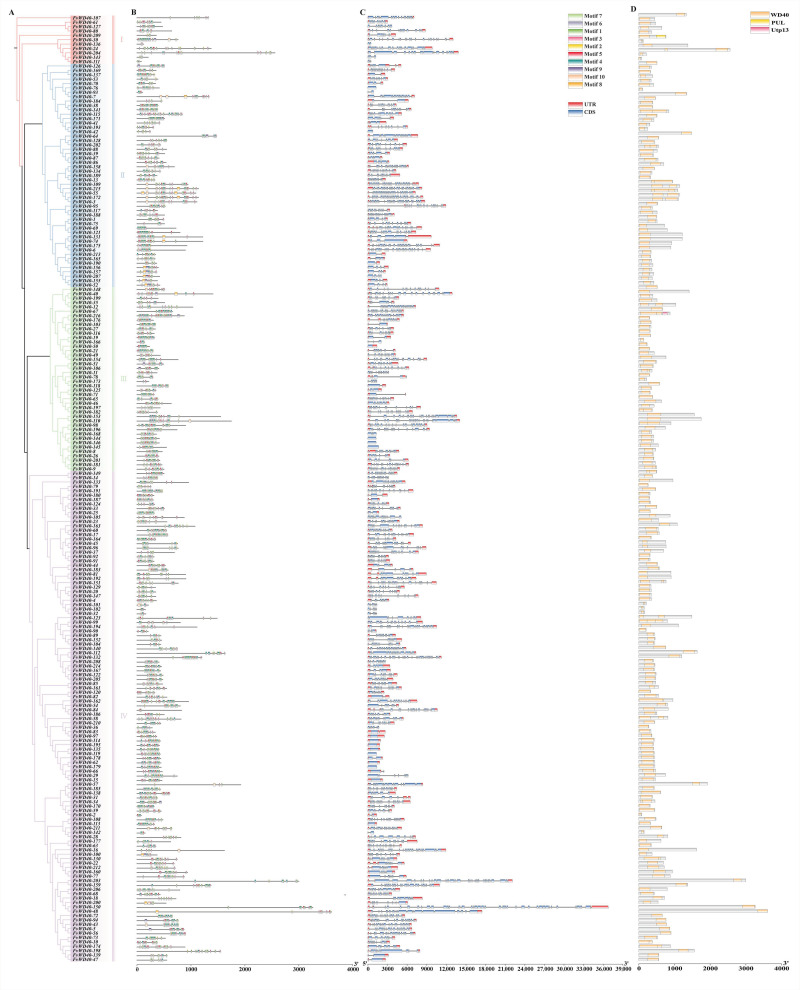
Motif and gene structure analysis of the *FvWD40* gene family. (**A**) A phylogenetic tree of FvWD40 family proteins. (**B**) FvWD40 family protein motif analysis. (**C**) Gene structure analysis of the *FvWD40* gene family. (**D**) Structural domain analysis of FvWD40 family proteins.

To further validate the diversity of WD40 in *F. vesca*, we analyzed the conserved motifs of the *FvWD40* gene family proteins. Motif 1, motif 3, motif 4, motif 5, and motif 7 are all widely distributed in the FvWD40 protein. Subgroup I does not contain motif 8. Only 17 genes contained motif 10, 16 genes contained motif 9, and 15 genes contained motif 8, all of which were contained more in the second subfamily (Figure 3B and Table S1).

We analyzed the exon and intron composition of *FvWD40* gene family members and found that 24 genes had no upstream or downstream sequences. Most of the genes contain more exons and introns, and only a few genes contain fewer exons and introns. *FvWD40-150* has the maximum number of exons with 39, while *FvWD40-42*, *FvWD40-47*, *FvWD40-62*, *FvWD40-76*, *FvWD40-139*, *FvWD40-144*, *FvWD40-145*, *FvWD40-146*, and *FvWD40-179* have only one exon (Figure 3C).

### 2.3. Chromosomal Localization, Subcellular Localization, and Covariance Analysis of F. vesca WD40 Gene Family

**Figure 4:**
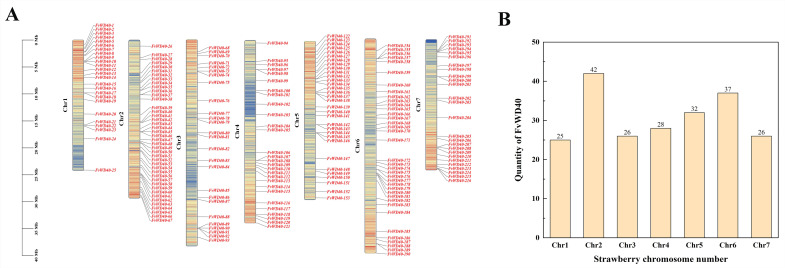
The chromosomal distribution of FvWD40 family genes. (**A**) The distribution of FvWD40 family genes on seven chromosomes. Different colors are used to differentiate the gene density on the chromosomes; where red regions represent the highest gene density, while blue regions represent the lowest gene density. The left scale indicates the chromosome length (Mb). (**B**) The number of genes distributed on each chromosome.

Chromosomal localization analysis of the *FvWD40* gene family revealed gene distribution on all seven chromosomes. The most genes were distributed on Chr2 with 42 genes and the least on Chr1 with 25 genes. 26, 28, 32, 37, and 26 genes distributed on Chr3-7, respectively (Figure 4).

**Figure 5:**
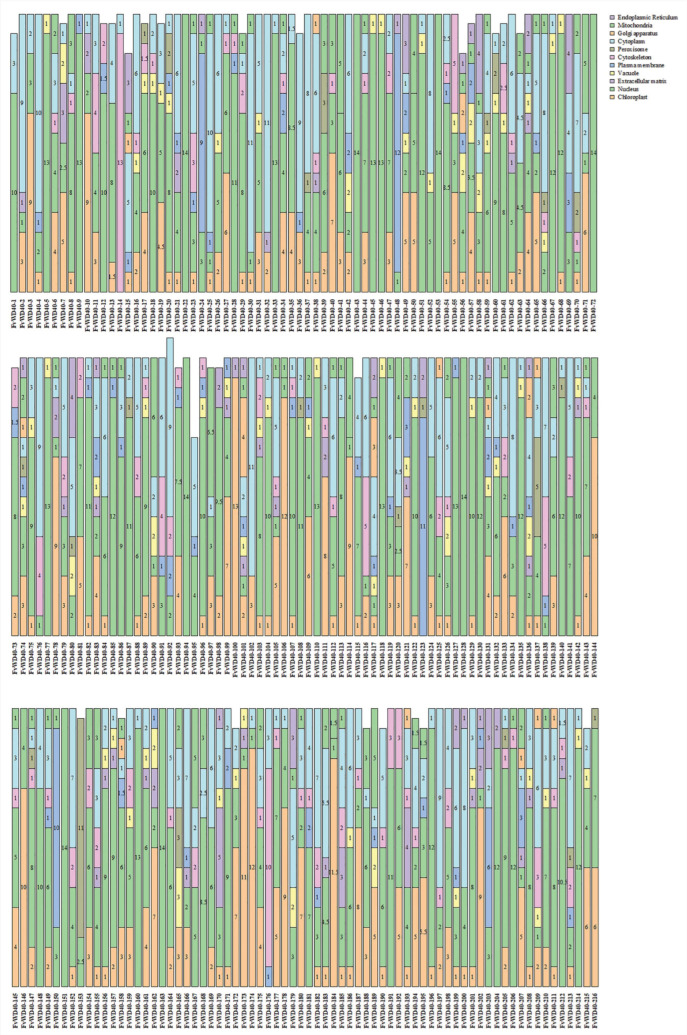
Predictions of subcellular localization of FvWD40 family proteins. The values on the bars reflect the frequency of gene localization in that organelle.

Subcellular localization results showed that the FvWD40 protein was mainly localized in chloroplasts, nuclei, cytoskeleton and cytoplasm. FvWD40-22, FvWD40-43, FvWD40-53, FvWD40-72, FvWD40-94, FvWD40-128, FvWD40-151, and FvWD40-163 are localized only in the nucleus. In total, 70 genes were localized in vesicles, 21 genes in peroxisomes, and 15 genes in the Golgi apparatus (Figure 5).

**Figure 6:**
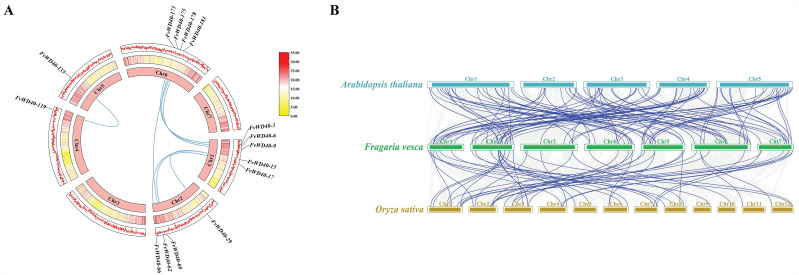
Covariance analysis of the *FvWD40* gene family. (**A**) Intraspecific covariance analysis of the *FvWD40* gene family. Blue lines indicate gene pairs with covariate relationships. The different colors represent the distribution of genes on the chromosomes, where the red areas imply high densities of genes, while the yellow areas imply low densities of genes. (**B**) Interspecific covariance analysis of the *FvWD40* gene family. Blue lines indicate covariant gene pairs between *F. vesca* WD40 family genes and *Arabidopsis* and rice.

Intraspecific colinear analysis of the *FvWD40* gene family revealed a total of eight colinear pairs, namely *FvWD40-3*/*FvWD40-173*, *FvWD40-6*/*FvWD40-175*, *FvWD40-9*/*FvWD40-181*, *FvWD40-15*/*FvWD40-29*, *FvWD40-15*/*FvWD40-66*, *FvWD40-17*/*FvWD40-60*, *FvWD40-62*/*FvWD40-178* and *FvWD40-119*/*FvWD40-135*. These data suggest that some members of the *FvWD40* gene family may have arisen by the process of fragment duplication, a key mechanism driving the evolution of the *F. vesca WD40* gene family (Figure 6A).

To further discover the affinities between *F. vesca* and *Arabidopsis* and rice, interspecies colinear analysis of the *FvWD40* gene family revealed a total of 100 colinear pairs between the *F. vesca WD40* gene and *Arabidopsis*, whereas 49 colinear pairs were found with rice, suggesting that *F. vesca* is more closely related to dicotyledonous plants, and is more distantly related to monocotyledonous plants (Figure 6B).

### 2.4. Promoter and Tissue-Specific Analysis of F. vesca WD40 Gene Family

**Figure 7:**
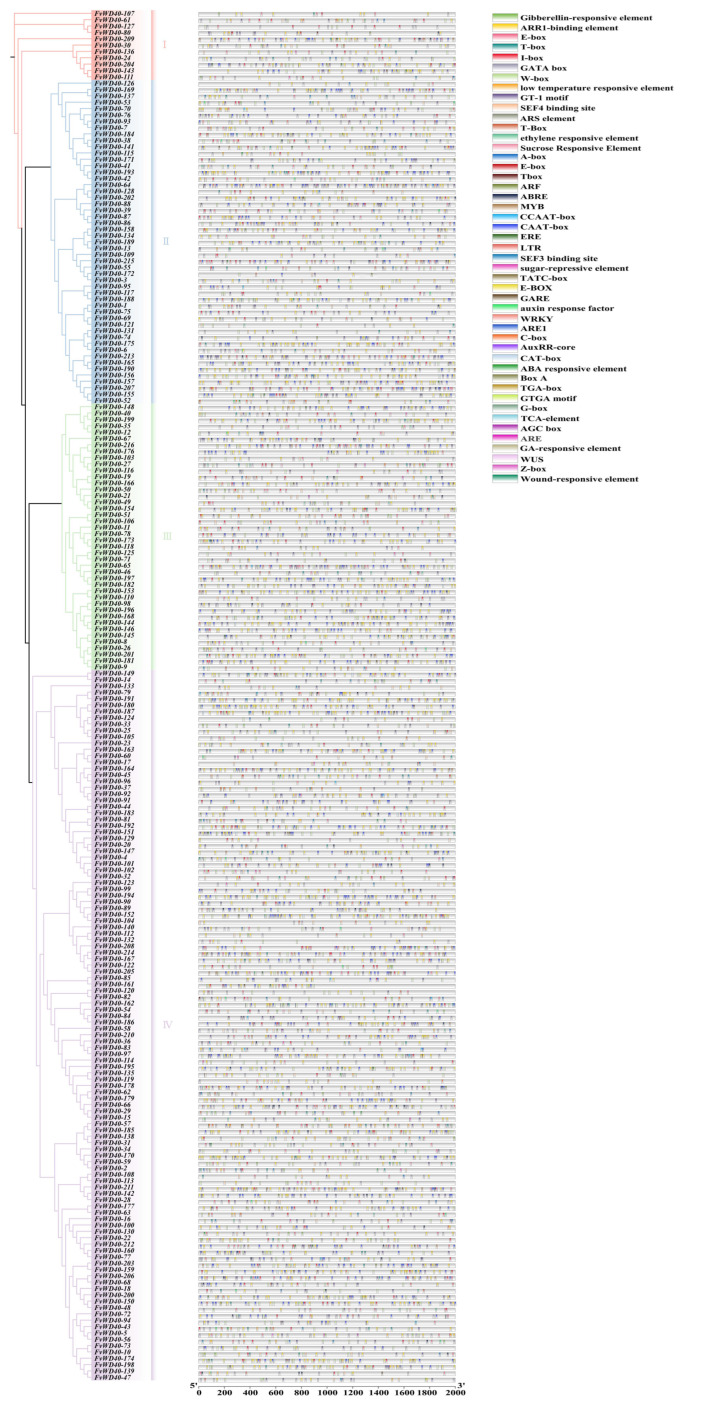
Analysis of promoter cis-acting elements of FvWD40 family genes. Different colored squares represent different function elements. Roman numerals represent different subgroups.

The first 2000 bp of the *F. vesca WD40* gene was analyzed for cis-acting elements, and the promoters of this gene family include light, hormone, and stress response elements. Light-responsive elements include elements such as G-box, I-box, E-box, and GT1-motif. Hormone response elements include elements such as TCA-element, ABRE, TGA-box, TATC-box, ERE, GA-responsive element, and AuxRR-core. Hormone response elements include elements such as WUS and LTR. WD40 family promoter sequences also contain MYB transcription factors and bHLH binding site elements such as E-box (Figure 7).

**Figure 8:**
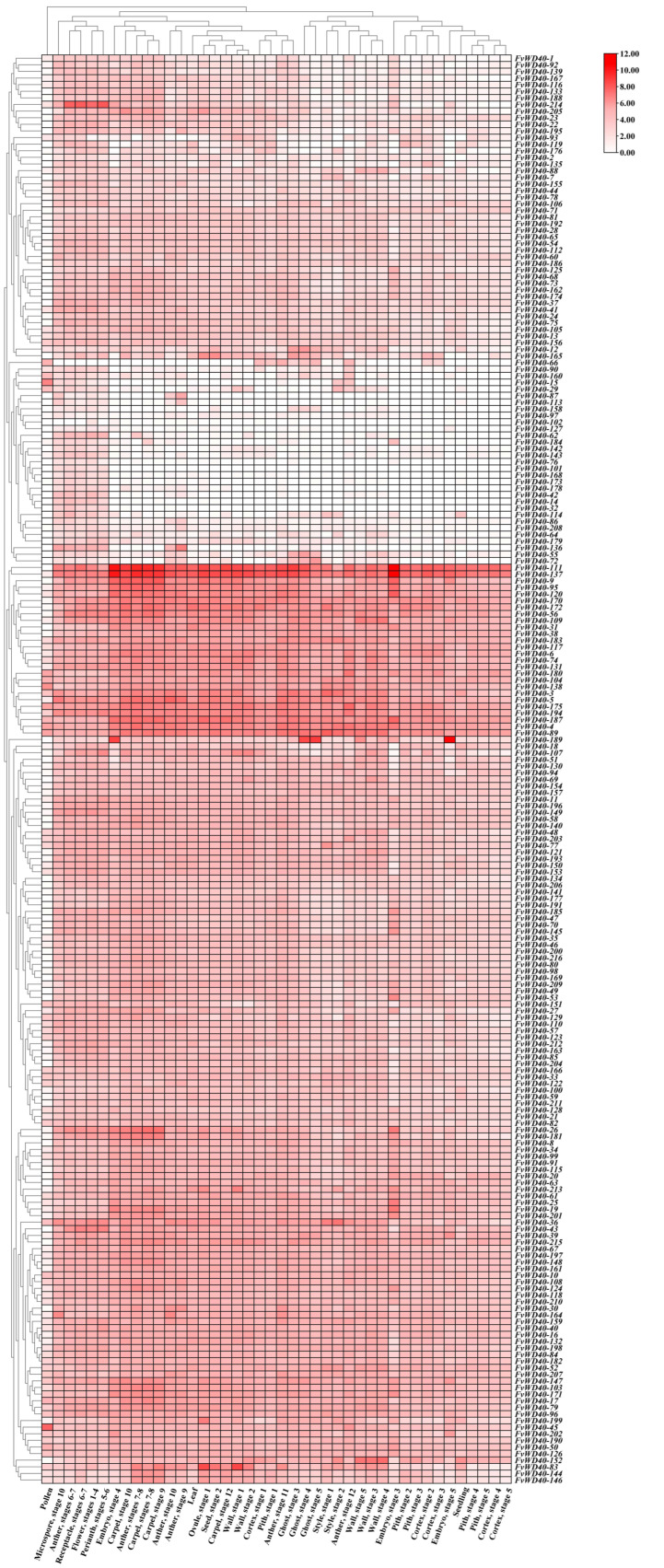
Tissue-specific expression analysis of the FvWD40 family gene. Different colors indicate the level of expression. The darker the color, the higher the expression. The lighter the color, the lower the expression.

By analyzing the expression of members of the *FvWD40* gene family in strawberry seeds, young leaves, seedlings, at different times of flowering, and in pollen, we found that *FvWD40-4*, *FvWD40-89*, *FvWD40-104*, *FvWD40-111*, *FvWD40-137*, *FvWD40-138*, *FvWD40-175*, *FvWD40-180*, and *FvWD40-187* were highly expressed in almost all tissues. *FvWD40-76*, *FvWD40-90*, *FvWD40-97*, *FvWD40-101*, *FvWD40-102*, *FvWD40-113*, *FvWD40-127*, *FvWD40-158*, and *FvWD40-208* were expressed at low levels in almost all tissues. The relatively high expression of *FvWD40-62* and *FvWD40-214* in flowers and *FvWD40-88* and *FvWD40-152* in exocarp suggests that these genes may have a potential role in pigment regulation (Figure 8).

### 2.5. Codon Preference and Selection Pressure Analysis of the F. vesca WD40 Gene Family

**Figure 9:**
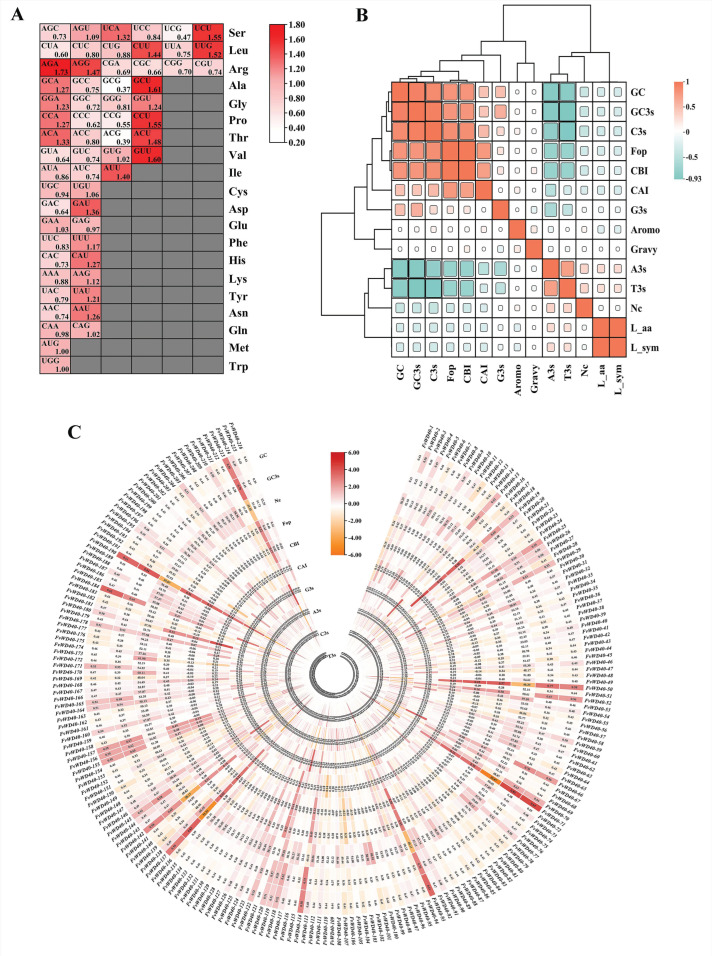
Codon preference analysis of the FvWD40 family gene. (**A**) Relative synonymous codon usage (RSCU) analysis of FvWD40 family genes. The gradient from white to red shows the ascending RSCU values. (**B**) Correlation analysis of codons in the FvWD40 family of genes. Orange indicates a positive correlation; cyan indicates a negative correlation. (**C**) Analysis of codon parameters of FvWD40 family genes. “A3s, G3s, C3s, and T3s” represent the frequency of occurrence of each base at the third position of the synonymous codon; “CAI” is used to measure the proximity of the codon usage preference of a gene to the optimally expressed gene; “CBI” reflects the bias of codon usage; “FOP” describes the frequency of occurrence of the optimal codon in a gene; “ENc” provides a measure of the diversity of codon usage; “GC3s” denotes the number of GC base pairs at the third codon position in a gene; and “GC” is the total number of GC base pairs in the entire gene sequence.

By analyzing the relative synonymous codon usage frequency of the *FvWD40* gene family, a total of 29 codons were found to have an RSCU ≥ 1, namely AGU, UCA, UCU, CUU, UUG, AGA, AGG, GCA, GCU, GGA, GGU, CCA, CCU, ACA, ACU, GUG, GUU, AUU, UGU, GAU, GAA, UUU, CAU, AAG, UUU, AAU, CAG, AUG, and UGG (Figure 9A). This indicates that Ser preferentially encodes AGU, UCA, and UCU, Leu preferentially encodes CUU and UUG, and Arg preferentially encodes AGA and AGG, among others. The mean values of CAI, CBI, Fop, and Nc for *FvWD40* gene family members were 0.208, −0.039, 0.4, and 53.261, respectively. A total of 22 genes were found to have Nc values less than 50, which were *FvWD40-19*, *FvWD40-47*, *FvWD40-50*, *FvWD40-56*, *FvWD40-70*, *FvWD40-71*, *FvWD40-84*, *FvWD40-93*, *FvWD40-107*, *FvWD40-114*, *FvWD40-137*, *FvWD40-139*, *FvWD40-142*. *FvWD40-143*, *FvWD40-145*, *FvWD40-149*, *FvWD40-169*, *FvWD40-180*, *FvWD40-190*, *FvWD40-192*, *FvWD40-200*, and *FvWD40-213*, suggesting that these 22 genes have strong codon preferences. There was a difference in GC content between members of the FvWD40 gene family within a wide area from 43.7% to 64.9%, whereas the variation in GC3s content was from 28.7% to 89.4%, with an average of 46.5% and 42.5% for GCs and GC3s, respectively (Figure 9C, Table S2).

Correlation analysis of codon-related parameters showed that GC was positively correlated with GC3s, C3s, Fop, CBI, CAI, and G3s, and negatively correlated with A3s, T3s, and NC. C3s, Fop, CBI, and CAI were weakly positively correlated with G3s. GC, GC3s, C3s, Fop, CBI, CAI, and G3s were weakly negatively correlated with NC and strongly negatively correlated with A3s and T3s (Figure 9B).

By analyzing the selection pressure on members of the *FvWD40* gene family with collinearity, we found that the ratios of non-synonymous substitution rate (Ka) to synonymous substitution rate (Ks) of these gene pairs were all less than one, which implies that this gene family mainly undergoes a process of purifying selection (Table 1).

### 2.6. Expression Analysis of FvWD40 Gene Family at Different Fruit Coloring Stages

**Figure 10:**
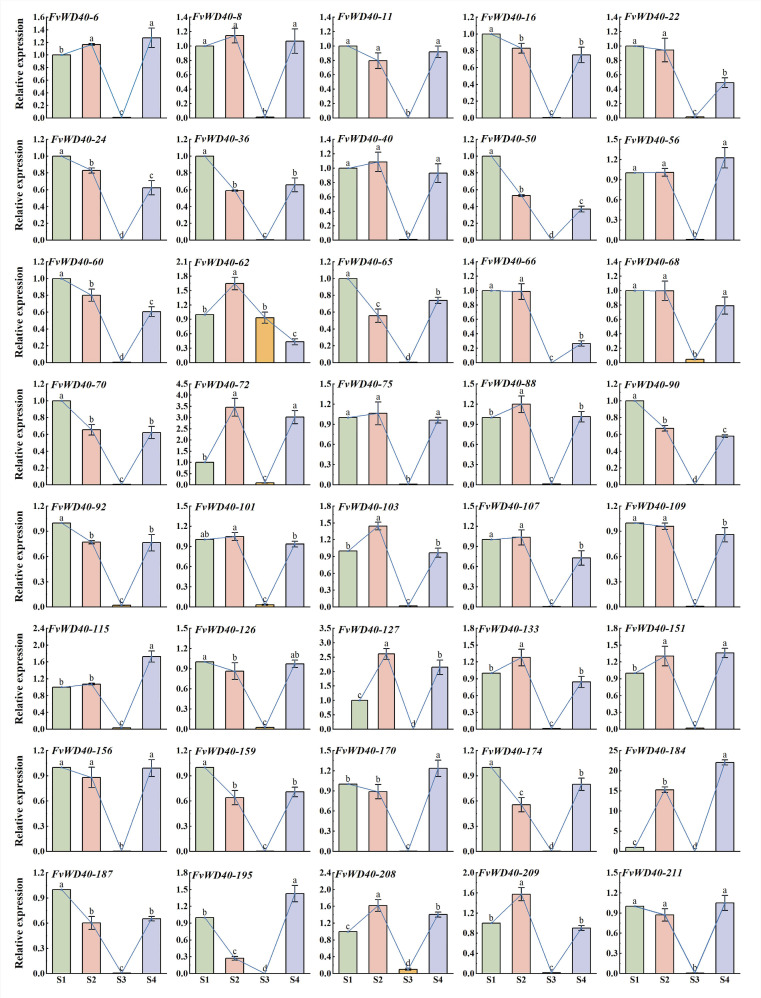
The expression of the *FvWD40* gene family at different stages of *F. vesca* fruit color formation. The S1 stage refers to the period when the fruit is white, the S2 stage refers to the period when the fruit has reached 20% color, the S3 stage is the period when the fruit has reached 50% color, and the S4 stage represents the time when the fruit is fully colored. After performing a systematic expression analysis, we used the 2^−∆∆Ct^ method to determine the relative expression levels of the *FvWD40* gene family at other fruit color formation stages, using the S1 stage as a benchmark. The error lines in the graphs show the mean values and their standard errors based on three biological replicates of the experiment. Different letter labeling allows us to observe significant differences between expression levels, while identical lowercase letters indicate that no statistically significant differences were found in these comparisons (*p* < 0.05). Line graphs represent trends in bar graphs.

In this study, we analyzed the expression of some family members of *FvWD40* at four different coloring stages of *F. vesca* and found that almost all genes were expressed at lower levels during S3. Compared to the S1 period, the expression of some genes was significantly down-regulated in the S2 and S3 periods, such as *FvWD40-24*, *FvWD40-50*, *FvWD40-60*, *FvWD40-90*, and *FvWD40-174*. The results suggest that these genes may be involved in the regulation of *F. vesca* pre-coloring. Some genes showed the highest expression in the S2 period, such as *FvWD40-62*, *FvWD40-72*, *FvWD40-88*, *FvWD40-103*, *FvWD40-127*, *FvWD40-133*, and *FvWD40-209*. The expression of *FvWD40-72* was 3.4 times higher in the S2 period than in the S1 period. The expression of *FvWD40-127* was 2.1 times higher in the S2 period than in the S1 period. *FvWD40-62* was firstly significantly elevated in the S2 period and then gradually decreased. *FvWD40-115*, *FvWD40-170*, *FvWD40-184*, and *FvWD40-195* had the highest expression during S4. The expression of *FvWD40-184* was 22 times higher in the S4 period than in the S1 period. The expression of *FvWD40-115* was 1.7 times higher in the S4 period than in the S1 period. These results suggest that the different members function at different stages of coloration, respectively (Figure 10).

## 3. Discussion

In this study, a total of 216 FvWD40 family members were identified. Compared with previous studies, there were fewer WD40 members in *F. vesca* than in *Arabidopsis* (230) [5], mango (315) [37], and wheat (743) [38], but more than in tomato (207) [7], potato (178) [39], and peach (220) [40].

To further validate the phylogenetic relationships of the FvWD40 family, we performed a phylogenetic developmental tree analysis, which can be divided into four subfamilies. Subcellular localization results showed that FvWD40 family proteins were mainly localized in chloroplasts, nuclei, and cytoplasm. Covariance analysis revealed a total of eight covariance pairs for FvWD40 family members, namely *FvWD40-3*/*FvWD40-173*, *FvWD40-6*/*FvWD40-175*, *FvWD40-9*/*FvWD40-181*, *FvWD40-15*/*FvWD40-29*, *FvWD40-15*/*FvWD40-66*, *FvWD40-17*/*FvWD40-60*, *FvWD40-62*/*FvWD40-178,* and *FvWD40-119*/*FvWD40-135*. *FvWD40-15* has two segmental duplications, and these results suggest that some *FvWD40* genes may have arisen from segmental duplications. Analysis of selection pressure on members of the *FvWD40* gene family revealed that the family members evolved by purifying selection.

Analysis of the promoter sequences of the *FvWD40* gene family members revealed that the promoters of the family members mainly contain response elements such as hormones and abiotic stresses. This is similar to the results for promoter cis-acting elements in tomato and in the *Oryza* genus [7,41]. In previous studies, light and hormonal components were found to be associated with anthocyanoside synthesis [20,42,43]. To verify the expression of FvWD40 family members in various tissues of *F. vesca*, tissue expression prediction was performed in this study. Some of the genes were found to be highly expressed in all tissues of *F. vesca*, and some genes were highly expressed in tissues where *F. vesca* is more pigmented, such as flower and peel. *FvWD40-62* and *FvWD40-214* were relatively highly expressed in flowers. In the study of Pachysandra, *HmWDR68* had the highest expression in sterile flowers and the lowest expression in roots [34].

The WD40 gene plays an important role not only in regulating plant response to abiotic stress and plant growth and development, but also in regulating anthocyanin and flavonoid synthesis [17,44,45]. Virus-induced silencing of the WD40 transcription factor gene reduced anthocyanin accumulation in chili fruits by decreasing *CHS*, *F3H*, *F3′5′H*, *DFR,* and *3GT* expression in fruits [46]. In apple, the apple WD40 protein (MdTTG1) interacts with bHLH to regulate anthocyanoside synthesis [30]. To further verify the role of *FvWD40* gene family in *F. vesca* fruit coloring, this study conducted analysis by qRT-PCR. *FvWD40-24*, *FvWD40-50*, *FvWD40-60*, *FvWD40-90*, and *FvWD40-174* were found to have the highest expression at the S1 stage, suggesting that these genes may play an important role in regulating pre-transformation staining in *F. vesca*. Similar results were seen in eggplant, where some genes decreased in expression as eggplant coloring increased, such as *SmWD40-27* and *SmWD40-43* [6]. Some genes had the highest expression during the S2 period, such as *FvWD40-62*, *FvWD40-72*, *FvWD40-88*, *FvWD40-103*, *FvWD40-127*, *FvWD40-133*, and *FvWD40-209*, suggesting that the expression of genes related to pigment synthesis is elevated during the early stage of strawberry fruit coloring in order to synthesize pigments. The lowest expression of basically all genes was observed at S3, which may be due to the fact that the expression of anthocyanin synthesis genes decreases as the plant may shift its metabolic focus from pigment synthesis to other ripening-related biosynthetic pathways as the fruits ripen. *FvWD40-115*, *FvWD40-170*, *FvWD40-184,* and *FvWD40-195* had high expression during S4, suggesting that these genes have a role in the regulation of pigmentation during fruit ripening and may also regulate pigment degradation. These results are consistent with the findings in eggplant [6]. Wang et al. [45] found that the expression of some genes decreased with the deepening of flower color at both bud and bloom stages in rhododendron, and some of the genes were most highly expressed in pink-colored flower species, followed by red flowers, and least in white flowers. Liu et al. [39] examined the peel and flesh of potato and found that most of the *StWD40* expression was lower in white peel and flesh, higher in purple peel and flesh, and a few genes were higher in white peel and flesh. At present, these genes involved in *F. vesca* fruit pigment accumulation and transformation have not been further validated and need to be further verified.

## 4. Materials and Methods

### 4.1. Plant Materials

The plant material used in this experiment was diploid forest strawberry “Hawaii 4”, which was grown in a light incubator at a temperature of 22 °C/19 °C (16 h during the day/8 h at night), a humidity of 50%, and a light of 7000 Lux. The cultivation substrate–vermiculite was 3:1. Fruits were collected at the white fruiting stage (S1), 20% coloring stage (S2), 50% coloring stage (S3), and full coloring stage (S4), the *F. vesca* flesh was separated from the pericarp, and the pericarp were collected, chopped, and accurately weighed to extract the RNA for the subsequent experiments.

### 4.2. Identification of FvWD40 Gene Family

We downloaded the *F. vesca* genome (Fvesca_677_v4.0) and GFF3 (Fvesca_677_v4.0) files from the Phytozome v13 (https://phytozome-next.jgi.doe.gov/, accessed on 20 April 2024) website [47]. Candidate genes were screened using Simple HMM Search of TBtools v2.136 [48], and structural domain screening was performed on the NCBI-CDD (https://www.ncbi.nlm.nih.gov/cdd/, accessed on 1 May 2024) and HMMER (https://www.ebi.ac.uk/Tools/hmmer/search/hmmscan, accessed on 1 May 2024) websites [49] to exclude genes that did not contain the PF00400 structural domain.

We analyzed the physicochemical properties of FvWD40 protein including molecular weight (MW), isoelectric point (PI), hydrophilicity, etc., using the ExPASy (https://web.expasy.org/protparam/, accessed on 1 May 2024) website [50].

### 4.3. Analysis of the FvWD40 Family Evolutionary Tree, Motif, and Gene Structure

Multiple sequence alignment of FvWD40 proteins was performed using ClustalX 1.83 software, and a phylogenetic tree with bootstrap value set to 1000 was constructed using MEGA 7.0 software. We beautified it at the Chiplot (https://www.chiplot.online/, accessed on 3 May 2024) website [51].

Conserved motif analysis was performed using the MEME (http://meme-suite.org/tools/meme, accessed on 3 May 2024) website [52], with the motif number set to 10 and the rest of the parameters at default values, and plotted the analysis using TBtools. Gene structures were mapped using TBtools via the GFF3 files.

### 4.4. Analysis of Chromosomal Localization, Secondary Structure, and Subcellular Localization of FvWD40 Genes

Chromosome localization analysis was conducted with TBtools. Secondary structure prediction was performed at the NPS@: SOPMA (https://npsa-prabi.ibcp.fr/cgi-bin/npsa_automat.pl?page=npsa_sopma.html, accessed on 4 May 2024) website. The online software WoLF PSORT (https://wolfpsort.hgc.jp/, accessed on 4 May 2024) [53] was used to perform subcellular localization prediction and secondary structure and subcellular localization mapping was performed using Origin software.

### 4.5. Analysis of Promoter Cis-Acting Elements, Covariance, and Selective Pressure in FvWD40 Gene Family

The sequence of 2000 bp upstream of the start codon of the *FvWD40* gene family was extracted on TBtools, analyzed on the PLACE (https://www.dna.affrc.go.jp/PLACE/?action=newplace, accessed on 24 October 2024) website, and mapped using the Tbtools software.

Genomic and GFF3 files of *Arabidopsis* and rice were downloaded from the Phytozome v13 (https://phytozome.jgi.doe.gov/pz/portal.htm, accessed on 6 May 2024) website for covariance analysis and plotted in TBtools software.

The Simple Ka/Ks Calculator method of TBtools was utilized to calculate Ka (non-synonymous substitution rate), Ks (synonymous substitution rate), and (selection intensity) Ka/Ks for the eight pairs of *FvWD40* genes with a covariate relationship.

### 4.6. Codon Preference and Tissue Specificity Analysis of the FvWD40 Gene Family

The codon usage characteristics of CDS sequences of the *FvWD40* gene were analyzed using the online software CodonW 1.4.2 (http://codonw.sourceforge.net, accessed on 9 May 2024), including relative synonymous codon usage (RSCU), effective codon (ENC), codon bias index (CBI), codon adaptation index (CAI), best codon usage frequency (Fop), T3s, C3s, G3s, T3s, C3s, CAI, CBI, Nc, Fop, GC, GC3s, L_sym, L_aa, gravy, and Aromo parameter correlation analysis. Tissue-specific expression analysis of the *FvWD40* gene family in the BAR database (https://bar.utoronto.ca/, accessed on 9 May 2024) was conducted. Plotting was carried out with TBtools. Clustering was performed using Cluster Rows and Cluster Cols in TBtools’ HeatMap.

### 4.7. Extraction of RNA from F. vesca Fruit

RNA was extracted from *F. vesca* fruits using a plant RNA extraction kit (RealTimes (Beijing, China) Biotechnology Co., Ltd.), and the quality and quantity of RNA was determined using a Poulton P200 microvolume spectrophotometer (Poulton Technologies, Inc., San Jose, CA, USA). RNA was stored at −80 °C for further analysis.

### 4.8. qRT-PCR Analysis

Primers (Tables S3 and S4) were synthesized by Bioengineering (Shanghai, China). Biological Engineering Co. RNA from *F. vesca* fruit was extracted and reverse transcribed into single-stranded cDNA as a template. Using the *FvGAPDH* gene as a reference, a 20 µL real-time quantitative PCR reaction system was constructed containing 1 µL of cDNA, 1 µL each of upstream and downstream primers (10 μmol/L), 10 µL of SYBR Green enzyme mixture, and 7 µL of ddH_2_O. The cycling parameters were 95 °C for 30 s, 50 cycles of 95 °C for 5 s, and 60 °C for 30 s. Melting curve analysis was performed after PCR cycling, and the program consisted of 95 °C for 15 s, 60 °C for 60 s, and 95 °C for 15 s. Three biological replicates were set up for this experiment. The relative expression of *FvWD40* gene family members was calculated using 2^−ΔΔCt^ [54].

### 4.9. Statistical Analysis of Data

Statistical data were analyzed using Excel software and were calculated and organized. Three replicates of qRT-PCR quantitative data were analyzed by one-way ANOVA using Duncan’s method with SPSS 22.0. Plotting was performed in Origin 2021.

## 5. Conclusions

In this study, 216 members of the *F. vesca WD40* gene family were identified and distributed on seven chromosomes of *F. vesca*. They were categorized into four subfamilies based on phylogenetic relationships. Promoter cis-acting elements revealed that FvWD40 family members contain many elements related to anthocyanin synthesis, such as growth hormone, gibberellin, and methyl jasmonate. Tissue-specific expression analysis revealed that some of the genes were more highly expressed in tissues with higher pigmentation content, such as in flowers and fruit peels. The qRT-PCR results showed that *FvWD40-24*, *FvWD40-50*, *FvWD40-62*, *FvWD40-88*, *FvWD40-115*, and *FvWD40-170* play roles in the early stage of *F. vesca* fruit coloration and in the stages of pigment accumulation and degradation, and these genes may be candidates for subsequent studies (Table S5). This study will provide an avenue for further research on the function of the *WD40* gene family in *F. vesca* coloration.

## Figures and Tables

**Table 1:** Selective pressure analysis of FvWD40 family gene.

Seq_1	Seq_2	Ka	Ks	Ka/Ks
*FvWD40-29*	*FvWD40-15*	0.305222801	4.37446151	0.0697738
*FvWD40-15*	*FvWD40-66*	0.303913669	2.018940614	0.150531257
*FvWD40-29*	*FvWD40-66*	0.302552807	2.025605656	0.14936412
*FvWD40-6*	*FvWD40-66*	0.8975621	3.141395351	0.285720834
*FvWD40-178*	*FvWD40-66*	0.68189289	2.742848067	0.248607606
*FvWD40-119*	*FvWD40-66*	0.553577916	2.663474605	0.207840508
*FvWD40-9*	*FvWD40-17*	0.866885205	3.533685587	0.245320412
*FvWD40-181*	*FvWD40-17*	0.785522234	2.549625771	0.30809315
*FvWD40-3*	*FvWD40-17*	0.894270532	3.180336765	0.28118737
*FvWD40-6*	*FvWD40-17*	0.977832084	2.218684428	0.440726077
*FvWD40-175*	*FvWD40-17*	0.971894622	2.199126665	0.441945722
*FvWD40-17*	*FvWD40-60*	0.189719651	2.179057226	0.087065015
*FvWD40-181*	*FvWD40-60*	0.889035953	2.482081889	0.358181556
*FvWD40-3*	*FvWD40-60*	0.850615298	3.54084111	0.240229728
*FvWD40-175*	*FvWD40-60*	0.928153321	2.655053714	0.349579866
*FvWD40-17*	*FvWD40-9*	0.866389302	3.35850378	0.257968833
*FvWD40-181*	*FvWD40-9*	0.127578955	1.43000704	0.089215613
*FvWD40-3*	*FvWD40-9*	0.907572081	1.910735214	0.474985793
*FvWD40-6*	*FvWD40-9*	0.974656242	2.96421201	0.328807872
*FvWD40-175*	*FvWD40-9*	0.890930831	1.505822225	0.591657379
*FvWD40-3*	*FvWD40-181*	1.039323568	6.901806751	0.150587173
*FvWD40-6*	*FvWD40-181*	0.937928334	3.13479313	0.299199435
*FvWD40-175*	*FvWD40-181*	0.785967165	2.21756012	0.354428797
*FvWD40-119*	*FvWD40-181*	0.903165816	2.677540657	0.33731171
*FvWD40-135*	*FvWD40-181*	0.984173969	3.928925522	0.250494432
*FvWD40-173*	*FvWD40-3*	0.415009419	1.54582695	0.268470814
*FvWD40-175*	*FvWD40-3*	0.84697515	2.056546415	0.41184344
*FvWD40-135*	*FvWD40-3*	0.986051527	2.294533154	0.429739499
*FvWD40-6*	*FvWD40-173*	0.955513315	3.353399352	0.284938719
*FvWD40-175*	*FvWD40-173*	0.972639509	1.738350417	0.559518668
*FvWD40-135*	*FvWD40-173*	0.786537794	3.684815489	0.21345378
*FvWD40-175*	*FvWD40-6*	0.094787717	1.421269733	0.066692279
*FvWD40-178*	*FvWD40-6*	0.907112019	2.559185413	0.354453419
*FvWD40-135*	*FvWD40-6*	0.957201603	2.405324578	0.397951117
*FvWD40-9*	*FvWD40-175*	0.889800726	1.616879521	0.550319745
*FvWD40-119*	*FvWD40-62*	0.615953751	2.474169827	0.248953707
*FvWD40-6*	*FvWD40-178*	0.909296212	2.58510592	0.351744277
*FvWD40-119*	*FvWD40-178*	0.656315239	2.411238577	0.272190087
*FvWD40-135*	*FvWD40-119*	0.311647823	2.508425684	0.124240405

## Data Availability

The species analyzed in this experiment was forest strawberry, and the forest strawberry genome version number was Fvesca_677_v4.0. Data will be made available on request.

## Supplementary Materials

The following supporting information can be downloaded at: https://www.mdpi.com/article/10.3390/ijms252212334/s1.
