# Supplementary material for: Genome-Wide Identification and Analysis of WD40 Family and Its Expression in F. vesca at Different Coloring Stages
Source: Int J Mol Sci. 2024 Nov 17;25(22):12334. doi: 10.3390/ijms252212334 (PMC11594367; doi:10.3390/ijms252212334)
Supplement: Supplementary file 1 [file ijms-25-12334-s001.zip › Supplementary Table S1.pdf]

Subgroup I contains motifs

Motif 1. LASGSDDKTVRIWDV

Motif 2. SGSDDGTVKVW

Motif 3. YLASASDDGTVRVW

Motif 4. KCVATLEGHTGPVTALAWSPD

Motif 5. VLKGHTGAVTSVAFS

Motif 6. SGSSDGTIKJW

Motif 7. HTAAVNSVAWSPSGN

Motif 9. LLHQIETJANPKGLCEVSQKPGPAVLACPGLQKGQIRVEHY

Motif 10. LKSSRLRTLINQSLNWQHQLCKNPRPNPDIKTLFVDHSC

Subgroup II contains motifs

Motif 1. LASGSDDKTVRIWDV

Motif 2. SGSDDGTVKVW

Motif 3. YLASASDDGTVRVW

Motif 4. KCVATLEGHTGPVTALAWSPD

Motif 5. VLKGHTGAVTSVAFS

Motif 6. SGSSDGTIKJW

Motif 7. HTAAVNSVAWSPSGN

Motif 8. NKVMIWDDHQSRCIGELSFRSEVRGVRLRRDRIVVVLEQKIFVYNFADLK

Motif 9. LLHQIETJANPKGLCEVSQKPGPAVLACPGLQKGQIRVEHY

Motif 10. LKSSRLRTLINQSLNWQHQLCKNPRPNPDIKTLFVDHSC

Subgroup III contains motifs

Motif 1. LASGSDDKTVRIWDV

Motif 2. SGSDDGTVKVW

Motif 3. YLASASDDGTVRVW

Motif 4. KCVATLEGHTGPVTALAWSPD

Motif 5. VLKGHTGAVTSVAFS

Motif 6. SGSSDGTIKJW

Motif 7. HTAAVNSVAWSPSGN

Motif 8. NKVMIWDDHQSRCIGELSFRSEVRGVRLRRDRIVVVLEQKIFVYNFADLK

Motif 9. LLHQIETJANPKGLCEVSQKPGPAVLACPGLQKGQIRVEHY

Motif 10. LKSSRLRTLINQSLNWQHQLCKNPRPNPDIKTLFVDHSC

Subgroup IV contains motifs

Motif 1. LASGSDDKTVRIWDV

Motif 2. SGSDDGTVKVW

Motif 3. YLASASDDGTVRVW

Motif 4. KCVATLEGHTGPVTALAWSPD

Motif 5. VLKGHTGAVTSVAFS

Motif 6. SGSSDGTIKJW

Motif 7. HTAAVNSVAWSPSGN

Motif 8. NKVMIWDDHQSRCIGELSFRSEVRGVRLRRDRIVVVLEQKIFVYNFADLK

Motif 9. LLHQIETJANPKGLCEVSQKPGPAVLACPGLQKGQIRVEHY

Motif 10. LKSSRLRTLINQSLNWQHQLCKNPRPNPDIKTLFVDHSC
