# Supplementary material for: Genome-Wide Identification and Analysis of WD40 Family and Its Expression in F. vesca at Different Coloring Stages
Source: Int J Mol Sci. 2024 Nov 17;25(22):12334. doi: 10.3390/ijms252212334 (PMC11594367; doi:10.3390/ijms252212334)
Supplement: Supplementary file 1 [file ijms-25-12334-s001.zip › Supplementary Table S3.pdf]

Supplementary Table S3. Primer sequences for FvWD40 family genes

Gene	Forward primer(5'-3')	Reverse primer (5'-3')
FvWD40-6	AGCGTGTCCGAACTGGATTATGG	GTCACCTCTCCACCAACACAGTAG
FvWD40-8	AGCGTGAACCAGTGCCAACC	GCTCCTCATCCTCACTATCTTCGTC
FvWD40-11	GTTGGAGCAGAGCCTCTACTTAGC	TAACATCCACAACTCCTCCGTAACC
FvWD40-16	CAGCAACGGACTCAGATTCAATCAG	CAGGACGCACAGCAGATGGAG
FvWD40-22	GCAACACGGAGAGCATCAACAC	TCTTTCCATCCCATCCAGCAGTTAC
FvWD40-24	CGCCTATCGTGGTCCTCTAATGG	TCAATGGAGTCTGCTGGCTTCTTAG
FvWD40-36	TGAACTGCCATCATTTGCTGAAAGG	TCCACCCACTTCCAACAGAAAGG
FvWD40-40	TATCCGCTGCCGTTGCCTTG	TGATGTTCTCTGCCTAGCCTACTTG
FvWD40-50	TCCTCCGACTCCCACTACATCTG	TCTTGACGCACTCGCCTGTTG
FvWD40-56	ACAGCAGCAACAACAGACACAAC	TCTTTCCTCGTACATCTTGGTAGCC
FvWD40-60	TCAACAAGAGCACCGTGGATGG	ACTATTGCCTAAGTCCGCCTCAAG
FvWD40-62	CTCCATCTCCTCCATTGCAGTCTC	CCGTATTCATTGTGGTCTGGTTGTC
FvWD40-65	GTGGCTGTGCTGCGAGGTC	GCTGATGCTGTTGTCCGATTCTG
FvWD40-66	GCGGCTCGGCTGATAAGACC	CGGCTCACGATCCTCCTCAAC
FvWD40-68	AGCAGCAACATCTATCCAATTCGC	CTGAACTGGTGGGAGAAGTGGTAG
FvWD40-70	TCAAGAGCCTCAAGCCGCATAG	CCTCACCAAGTTAATCATCGCCAAG
FvWD40-72	GGGATACCAGGTGGAGATTTGAGTC	GCTTGAAGTGCTTGCCAGTGAC
FvWD40-75	TAAGGCGGAACATTGATGAAGTTGC	CCCAGTTTCCAGATCCACCAGAG
FvWD40-88	GTGTTGGCGGAGAGCGAGAG	CCTTTAAACCCAGCTTCATGTCCAG
FvWD40-90	CTGGGCACTTGGATTTCTCGTTTG	CAGCAACTGACTTGGATAGGTTTCG
FvWD40-92	TGACAATCTCACGGCTCACACAG	CCATAGGCTGACCAAGGAATCGG
FvWD40-101	CACTGGTCACTCTTCACTCCTCTG	GGCCATGCACGAAATATCCAAGAC
FvWD40-103	GGCTGGCAGTTCTGATAGGATGG	TGGAAGACAGACTCATTGACGGATC
FvWD40-107	GAGGCGGCAATTATCACTGAATGG	AAATACGAGCAGCACACCAAGTAAC
FvWD40-109	GCCGTGGAGATACTGGTGAAGG	AAGAGTCAACAGATGCGTGATTTCC
FvWD40-115	AGCAGCACACCATAGAGAAGAATCC	CAACACAACAGGAACCGCATCAG
FvWD40-126	AGCAAGTTCTCAAGTCGTCACATTG	GTCCATCCATACTGAAGCCGAGAG
FvWD40-127	GAACAAGCCTCAACAGCCTCTTTG	ACCGCCAGCAAGATATACACCATC
FvWD40-133	AGCGAGTGGTCTATGCGATTGG	CAACGGTGAAGATGCGGATGAAG
FvWD40-151	TAGTGGAAGTGATGATGGCAGATGG	ACGACACAATCAGAGGGATGACAC
FvWD40-156	AGTCTGGCGTGCTTTGCTCTC	TGCTCCTCTCCTCACCTCTTGG
FvWD40-159	ATGTGCTCCTCTGCTTGCTGAG	TCATCTCCACCATTGCCTTCTCTAC
FvWD40-170	ACTTGCGACGGCGAGCTATG	CAGGGTATTGGATGGTGCGGTAG
FvWD40-174	TGGGAGTGGCGGTCAGAGAG	GAATCAGGCGAATAGGCAACACAG
FvWD40-184	GGCGGACTCAACTTACTCAGGAC	AAGCCTCGTGTGCGTGTACC
FvWD40-187	CACCACTAGGATAGACCAAGCAGAC	TGTATTGTCGCAGCCACCAGAG
FvWD40-195	CTCCATTTCGTGCCTGTCTTTATGC	CCGCCTTCACCGAGCCATC
FvWD40-208	GGACCTGTCTGTGGATTGGAGTG	TGATGTGCTCGCTCGGACTATAAC
FvWD40-209	GTGTTGTGCTGCTGTCTTCAAGG	ATGGGCACTTCTGTAACCTCATACC
FvWD40-211	TCCACCCGCAATTACATCTCTCAG	CCAGTTATTTGTTGACCCGCAGAC
FvGAPDH	CATTCATCACCACCGACTACA	GAAGGGTCTTCTCATCCTTGAC
