# Supplementary material for: Genome-Wide Identification and Analysis of WD40 Family and Its Expression in F. vesca at Different Coloring Stages
Source: Int J Mol Sci. 2024 Nov 17;25(22):12334. doi: 10.3390/ijms252212334 (PMC11594367; doi:10.3390/ijms252212334)
Supplement: Supplementary file 1 [file ijms-25-12334-s001.zip › Supplementary Table S5.pdf]

>FvWD40-24

ATGGAGGAGAAGAGCGGCTCCTCATGGCGTCTGCAGAGTGGTTCATATCTGGGAGAAG
TCTCGGCCCTCTGTTTCCTTCACCCACCTGCTCACCTCTCTCATCAGCTTCCTTACTTGG
TGGCCGGGTCGGGTTCGCAAATCTTGGTCTATGAATTGGAGCAGGGAACTATGTTGAG
ATCCTTGGACGTTTTCCAAGGCATTCGCGTGCAAGGCATTTGCTGCGGCAGCGGCGCT
GTGATTGGCGACGACGGAAGTATTGGTTTCGATATGGCTGTGTTTGGGGAGAGGAGAG
TGAAGATGTTTAGATTGGAGATTGATTTGGGGCAGCAGCAACAAGTCTTGGATGTTTGT
TTGAGGCTGTTGCAGTTGTTGCCTAGCTTTGGGAACTGGGTCTTGGATGTTTCCTTCAT
CAAGCATGGGGGAGGTGAATGTGTTGCTGTTGGGTGCAGTGACAACTCTGTTCATGTT
TGGGATGTAGCTAGTTGTAATGTGGTTCTTCATGTTCAGCATCCTGAGAGGACTCTTCT
GTATTCAATGCGGTTATGGGGTGAAACTCTTGAAGCTCTGCGCATTGCATCCGGTACTA
TCTATAATCAGATCATTGTTTGGAAAGTGGCTCCTGAGTCTGAAGCATCATGTTTGACA
AGCCAAGTTGAACACCGTATTGATCAAAGTAATTCACTCTCAAATGGTGTTCAGCTTCC
TAATTGTCAGTATGAAGTTATACATATCAGCAAACTTGCAGGACATGAAGGTTCAATATT
TCGCCTATCGTGGTCCTCTAATGGATCAAAACTGGTTTCCGTATCTGACGATCGTAGTGC
TCGTGTCTGGGCAGTTTGTACTGAAACAAAGCATTCTAAGAAGCCAGCAGACTCCATT
GAACTTATGTTGTTTGGTCACAGTGCCCGAGTTTGGGACTGCTGCATTCTTGGATCGTT
AATTGTCACAGCTGGTGAGGATTGTACTTGTCGCGTGTGGGGGCTGGATGGTAAGCAC
CTGGAGACAATCAAGGAGCACACAGGAAGGGGCATATGGCGATGTTTGTATGATCCAA
AATCTTCGCTTCTAATTACTGCGGGCTTTGACTCTGCAATTAAAGTACATCAGCTGCACA
TTTCATACTCTGGGGGATTAGACGGACTTGCAGAGACAAAACAAATTGATGGAATATTT
ACATACACAACTCGTATCCCTACATTATGTGAGAATATTGGACCTATGGACAGCAAAAG
TGAATATGTTCGTTGCTTGCGATTTACATGTGAGGACACCCTTTATGTTGCCACAAACCA
TGGTTATTTGTACCATGCTAAGCTATTGGATACTGGAGAAGTAGAATGGACGAAACTCG
TTCGCGTCAGTGACGAGGTCCCAATTGTTTGCATGGATTTATTGTCAGAATCATTCAATC
TTTCTTCGGGTGTTAAGGATTGGATCGCTGTTGGAGATGGTAAAGGAAACATGACAGT
TGTTGGGGTTATGTATGGTGCTTCTGCCCCAAAAGTGGGTTTTGCTTTTACTTGGTCAG
CTGGAAAAGAAAGACAACTCCTTGGAGCACATTGGTGCCAATCAGTAGGTTATGGGTA
CATCTTCACTGCTGATCATAGAGGAACGCTGAAGCTCTGGAGTCTGTGTCACTGCTCTG
CAAAGAGCTGTGATGTGTCTCTCTTAGCTGAATTCACATCAAGTTTCGGAAGTAGAATT
ATGTGTTTAGATGCATCATTAGAGGAAGAGGTATTGGTATGTGGAGATATACGTGGAAA
TCTTCTTTTGTTCCCTTTGTTGAAGAGTGTGTTGCTGGGCACATTGGTTGCTGATGATAA
CATATCTCCATCAAGTTGTTTCAAAGGTGCTCATGGGATATCAAGCATCTCCAGTGTTGC
TGTTGGTAGACTAAGTTCTAATCAGATAGAAATATGCTCGACAGGAGCAGATGGGTGTA
TATGCTATCTGGAATATGACAAAGATAGGAAGGATTTGGAATTCATAGGGATGAAACAA
GTGAAGGAGTTGAGTCTTATTCAATCTGTCTCTGCGTGCAATAGCTCTGTGACCAAGCT
GTCAAATTCTCGTTACGCTGCTGGTTTTGCATCAGTGGATTTTATAATTTGGAATTTATTA
ACTGAGACCAAGGTCATCCAAATTCCATGTGGTGGATGGCGGCGTCCTCATTCCTATTA
TCTTGGTGATGTACCAGAGATAAAGAACTGCTTTGCATATGTTAAGGATGATATCATTTA
TATTCATCGACACTGGGTACTGGATGGTGACAGGAAGGCACTCTCTAGGAATTTGCATA
TGCAATTCCATGGGAGAGAGATGCATTCCATATGCTTTGTCTCAGAGGAATTGCAACAT
GGAGTAATTGGGAAGGACCGTCTGTCCAATAGATCTAGTTGGATTGCAACTGGTTGTG
AAGATGGAACTGTCAGGTTGACTAGGTATATGCCAGGTGTTGAGAATTGGTCTGGATCA
AAATTGCTTGGGGAGCATGTTGGTGGATCAGCTGTGAGATCAATATGCTCTGTGTCAAA

GATAAACATACTTCCATCAGATATGACGAGCTATCTCAATATGAGAACTCGTGACAATG
AAGCAACAGAGAATAGAGAAACTCCGGCTTTATTGATTTCTGTTGGTGCAAAGCGGGT
TTTGACTTCATGGCTACTAAGAAATAGGAAAGTAGACAAGAAGGAAGAAATAGTATGT
GATCTGCAGCATGACAACACTGGAAATGGCAATACATGTCTGTCCCCAGAGTCACCTT
CAATGTCATTCCAGTGGCTATCTACAGATATGCCAGCAAAATATTCTAGTATTCAAAAAG
TTCCAAACATTGAGAAAAGAGTTGATCAAGCTGGGGACGTTTCTGATGGGAAAGATGC
AGCATCAGAAAAGGGAAACAAAGAGTTGAACCTCATTAAGGATAAATATGAAGATGAC
TGGAGATACATGGCTGTTACTGCCTTCCTAGTGAAATGTGTCAATTCCAGGATAACCGT
CTGTTTTATTGGTGTCGCTTGTTCTGATGCCACCCTTGCATTACGGGCTCTAGTTTTGCC
TTATAGGCTATGGTTTGATGTTGCTTTTCTGTGTCCTCTATCATCACCAGTTTTATCCCTA
CAGCATGTCATCCTTCCTGCATGTCTACCTTCTGAAGGCAATTGGCAGATTGGAAGTTT
GTATATCCTCATCAGTGGGGCTACTGATGGAAGTATTGCCTTTTGGGACCTGACTAAAA
GCATTGAAGCTTTTATGCAGCTGGTATCAGTACTTGATGTAGAAAAGTTCATTGACTGT
CAGAAACGGCCGAGAACAGGGAGGGGAAGTCAAGGTGGACGATGGTGGAGATCTCT
AGGCAGTAGCATGTCCCGGAATAGGCAAGGTGCCAGTTCCACCGCTGTTAAAGCTGGA
GTGGGAACCGATGAAAAGCCAAAACATTCTGGAACTTCATCAATGCTAAATGATCATG
GGAGCAGTAGAACGGCTTCTTCACATGCTACTCATACTGCTTCACTAGATTCAGAAACA
AGTGCTTATGATTCCTCATCGGACATATGTGAAATATCACCTTTATTTGTCTTCAAGGCC
ATTCATCTATCTGGTGTCAATTCTCTCTATGTTTCAGATGTAGAGGGTTGTCAAAGTCCT
GAAATCGGTTTTCTCTATAATTTGATTAGTGGGGGTGATGATCAAGCCCTTAGTTGTCTC
ACATTTGAATTATCAGTGTCAACATCAAGCTCTGAATTTGATAATATGACACTGGAAATA
AAAAATTCAATCTCTGAATCTGGAAATGCAAAGAAGTTAATTCATTGTAACCAAGACA
AGAACTATTGGATCCGATTCTTAAATCATGATAAAGTCCCCTCAGCTCACAGCTCTGCC
GTAAAAGGTGTTTGGACAGACGGATCTTGGGTTTTTTCAACTGGTCTAGATCAGCGTG
TCAGGTGCTGGCGTCTCCAGGAGGAAGGTAAATTAATCGAGTATGCTTATCTGGTCATC
AGTGTGCCAGAGCCAGAAGCTCTGGATGCTAAACTGTGTGGCAGGAACAAATATCAGA
TTGCAGTAGCTGGGAGGGGAATGCAAATGCTGGAGTTCTCTGAAATATCTGGCATCAG
TTAA

>*FvWD40-50*

ATGGCGAGCAGCGGCAGCGGACAACAACTGCCGCCGTACAACCCTTACCGGCATCTG
AAGACTCTTCGCGGCCACGAGAGCGCCGTGTCGTGCGTCAAATTCTCCAACGACGGG
AACCTCTTGGCCTCGGCGTCGTTAGACAAGACCGTAATCCTCTGGTCGTCTTCCACCCT
AACCCTCCTGCGCCGCCTGGTGGGCCACTCCGAGGGCATTTCCGACCTTTCCTGGTCC
TCCGACTCCCACTACATCTGCTCCGCCTCCGACGACCGCACCCTCCGCATCTGGGACG
CACGCTCCCCAACAGGCGAGTGCGTCAAGACCCTCCGCGGCCACTCCGACGTCGTTTT
CTGCGCCAACTTCAACCCCCAGTCCAACCTCATCGTCTCCGGCTCGTTCGACGAGACC
ATCCGGATTTGGGAGGTCAAGACCGGAAAGTGCCTCAAGGTCATCAGGGGCCACACG
CTTCCGGTGACCTCCGTGAGCTTCAATCGCGATGGGTCGCTCATCGTCTCCGCCAGCCA
CGACGGGTCCTGCAAGATTTGGGACACGGAAGGGACCTGCCTCAAAACTGTAATTGAT
GATAAAGACGATAAAGCCCCTGCTGTTTCCTTCACCAAGTTCTCCCCTAACGGCAAATT
CATACTCATCGCCACTCTTGATAATACCCTTGTGAGTCCAAGTTCTCCCTTTTTGATTTA
G

>*FvWD40-62*

ATGGAGTTAGAATTCTACCGCAGGAATTCTCTGTTTCGCTTAATAGACACCGAAAGAAA

TATTGTACTAGAGTCGCCCAATGACCTCCATAACATCAACACCAATGACGAAGTGTATG
ATGATGAAGATCTCCAATTCAGCCCTTCAATGTCTTCAAGCCCCTACATAACTACAATGC
TTACACTAATGCCACCCCCAAGCCCGGAGTCCCCATGGACCCTCTCCCCTCACCAAAC
CCCATCTCCTTCACTCCTTTACCAATGCATTGCATCCCTCTATCGCCCCGAAGGCTCCAT
CTCCTCCATTGCAGTCTCAAAGCAGCAAGGAGTGGTCTTTGCTGGCTCAGAGAGTACC
CGAATCCATGTGTGGAGACAACCAGACCACAATGAATACGGATGTCTCAAGGCTACTT
CAGGTGAGGTACGAGCCATTGCAGTTCATGGCAACATGCTCTTCACTGCACACAAAGA
CCACAAAATCCGAATGTGGAACTTCACGGTCACAGATCACATTTTCAAGTCCAAAAAG
GTCTCTTCTTTCCCTAAAACAAGCTCGTTCACTTTATTTTCAAGATTTACAAACACCAA
ACAGCAACACAAAGAATGCATTTCCTGCTTGGCATATTATCAAACCGATGGTCTTGTCT
ATACTGGCTCCCATGACAGAACTGTCAAAGCGTGGCGACTTATAACCAATCACTGTGTG
GACTCATTTGTGGCGCATGAAGACAATGTGAATGCAATAGTGGTGAACCAAGATGATG
GGTGCGTTTTCACTTGCTCCTCAGATGGTTCTGTCAAAATCTGGAGAAGGGTATTCAGA
GAAAACTCTCACACTCTCACCATGACTCTCAAATCCCAATTCCCAAATTCCCACCACCC
TATAAACGCCATAGCCTTAAACGTATCATCCAACTCTTGCTTCCTCTACTCTGGTTCTTC
AGATGGAACCATTAACTTTTGGGAGAAGGAGAAACTGACACACAGGTTTAACCATGGT
GGGTTTTTACAAGGCCACCATTTTTCTGTTATGTGCTTAGTGGCCATTGAGAAGTTGATA
TTCAGTGGATCAGAGGATACTACAATTAGGGTTTGGAGGAGGGAAGAAGAGGGTTGTT
TTCATGAGTGTTTGGCTGTGTTAGAGGGGCATAGAGGCCCTGTGAGGTGTTTGGCTGC
TTGTTTAGAGATTGACACAGTTGTGAAAGGGTTTCTGGTTTATAGTGCGAGTTTGGATC
AGACTTTTAAGGTGTGGAGGGTTAAGGTCTCGCCGGAAGAGGGAATGTGGTTGAACT
CTCATATGGATCGGAGGAACAATGGGATCGAGGATGGAATGTGA

>FvWD40-88

ATGCCCACGGAAACCAGAAGAATGGCGTTCCCGAAGGTGGTGATCGAGCGCGACACC
GACTCCGAGCAAAGCTCGTCGGAAGAAGAAGAAGAAGACGACGAACCGGCGGTGTT
GGCGGAGAGCGAGAGTGAAGAGAAAGTTGAGGAGCCCAAGAACAAAGGGAAAGCC
CCCATTACTATTTCTCTCAAGAAAGTCTGCAAGGTTTGTAAGAAGCCTGGACATGAAGC
TGGGTTTAAAGGGGCTACTTACATTGATTGCCCAATGAAACCTTGCTTTCTTTGCAAAA
TGCCTGGCCACACTACAGTGACATGCCCACATCGAGTAGCTACCGAGTTTGGTGTCGT
CCCAGCAACACATAAAAATACCAGAAATGCATTGGAATATGCTTTCGAACGACAGATTA
GACCTCGGATTCCTCCGATCAAGCCAGCATATGTGATCCCTGATCAAGTAAGTTGTGCG
GTTATCAGATATCACAGTAGACGAGTAACTTGCTTGGAATTCCATCCAACAAACAATAA
TATCCTTTTGTCAGGAGATAAGAAAGGACAGCTTGGAGTCTGGGACTTCCGCAAAGTA
TATGAAAAGGTTGTTTATGGAAATGTGCACTCATGTATACTCAACAATATGAAGTTTAGC
CCTGCAAGTGATGGTACAGTATATGCTGCGTCCTCAGATGGTACTGTTAGTTGCACTGA
TGTCGAAACTGGAATTGCATTGTCTCTGATGAACCTTAACCCTGATGGTTGGCAGGGTC
CAAGCACCTGGAGAATGCTGTATGGCATGGATATAAATGCAGAGAAAGGTGCTGTACT
TGTTGCTGATAACTTTGGTTACCTCTACATGGTTGATACTCGGTCAAATAACAGAACTG
GGAAACCAATTTTGATCCATAAGAAAGGTAGCAAAGTTGTTGGACTCCACTGCAATCC
TGTTCAACCAGATCTTCTCTTGAGTTGTGGAAACGATCATTTTGCTCGCATATGGGACA
TTCGTCGAATTGAAGATGGTTCCTCCATTTATGACCTTGCACACAGCCGTGTTGTTAAC
TCTGCATACTTCTCTCCAGTATCTGGCAGTAAAATTCTCACCACTTCACAGGACAACCG
CCTTTGTATTTGGGATTCCATATTTGGCAATATGGATTCCCCAAGCCGAGAGATTGTACA
CAGTCATGATTTTAATCGCTATCTGACTCCTTTCCGAGCTGAATGGGATCCAAAGGACT

CGTTAGAGTCACTTGCAGTTATTGGTCGTTATATAAGTGAAAACTATAATGGAGCTGCTC
TGCATCCCATTGATTTCATAGACATCAGTACAGGGCAACTAGTTGCTGAGATCATGGAT
CCAAACATCACTACAATCAGTCCGGTGAACAAGCTGCATCCACGTGATGATGTTTTGGC
ATCCGGTAGCTCAAGGTCACTTTTCATTTGGAGGCCTAAGGAAAAGTCTGAGCCTGTG
GAACAGATGGATATAGGGAAGATTGTTGTTTGTGACAGAGATGAGAAAAAGAGTAGAA
AACGGAAGTTTGGGGGTGAAAGTGATGATTCTGATGATGACAAGTCCATCCCTAAGGG
CAAGCAGTTCAAGTCCCAAAAGAAATCTCAGTCCAAAACAAATCTATCTGTTAAGGTC
AGACGCTGA

>FvWD40-115

ATGTCGTCCGCCTTCAACGGTGGCCAGACTGACGTCGAAGTAAAGCGAGTGTTCATAG
GAGCAGGGTGCAACAGAATAGTGAACAACGTCTCATGGGGTGCTTGTGATTTAGTCGC
TTTCGGCGCCCAAAACGCCGTCGCTGTTTTCGACCCAAAGACTGCTCAAATTTCGACT
ACGCTTCCGGGCCACAAGGCCTCTGTTAACTGCACCCAATGGCTTCCAAGTAATAAGT
TTGCATTCAAAGCTAAAGACTTAGACCAACATTACTTGCTTTCTGGAGATGCTGGGGGT
GCCATTATTTTGTGGGAGTATACTGTTCTTGAAGGAAAGTGGAGATACGTACTGCAAAT
ACCTGAATTACACAAGAAGGGTGTCACATGCATTAGTGGGATTTTGGTTTCCGATACTG
AGGCAGTCTTTGCTTCTACTTCGTCGGACGGTACAGTTTATATCTGGGAGGTTGTTTTC
CCAACTACTGGTGGAGGTGACTGTAAATTATTGCATTTGGATTCTCTATTTGTTGGTTCA
AAACCTATGGTAGCACTTTCACTAGCAGAGTTGCCTGGGAATACTGGGCATCTAGTCCT
GGCCATGGGGGGATTGGATAACAAGATTCACCTATACTCTGGGGAGAGGAGAGGAAAG
TTTGTTCGAGCCTGCGAGTTGAAAGGACATGCAGATTGGATCAGAAGCCTGGATTTCT
CGTTACCTATTTTCAATAATGGTGAGGCACATAATATTCTACTTGTTAGTTCATCACAAG
ACAAAGGGATACGCATATGGAAGATGGCTCTACGGGGTTCTTTGGACAGCAGCCAGAG
TTCCAAGCCAGGAAAAATAAGCTTAGCATCTTACATAGAAGGCCCTGTACTTGTCGCTG
GAACAACCTCATATCAGATTTCACTGGAATCTCTTCTAATTGGTCATGAGGATTGGGTGT
ATTCAGTGGAGTGGCAACCACCTTCACCTGTATCTTCAGACGGGATTGCGTACTGTCAA
CACCAGAGCATCTTATCTGCATCTATGGATAAGACAATGATGATCTGGAAACCAGAAAA
AACTTCTGGTATCTGGATGAATGTTGTTACTGTTGGGGAATTGAGTCATTGTGCTCTAG
GATTTTATGGTGGCCACTGGAGTCCCAACGGAGATTCGATCTTAGCACATGGGTATGGT
GGATCTTTCCATCTGTGGAGAAATGTCGGTACTGGCTTAGATAATTGGCAACCGCAAAA
AGTTCCATCTGGGCATTTTGCAGCTATTACCGATATTGCTTGGGGGAGATCTGGTGAATA
CTTGCTGTCAGTCAGTGATGACCAGACAACACGAATTTTTCTCCATGGCAAAATGAA
ACATCTCTAGGAGATGAGGGTTCTTGGCATGAAATTGCTCGTCCTCAAGTTCATGGTCA
TGATATGAACTGTGTCACCATCATCCAAGGAAAAGGAAACCATCGTTTTGTCAGTGGA
GCCGATGAGAAAGTTGCCAGAGTGTTTGAAGCTCCTTTGTCTTTCTTGAAGACATTGG
GTCATGCCATTTCACAAAACTCTACCTTTTCTGAAGATATTCAGTTGGGTGTACAGATAT
TGGGTGCAAATATGTCAGCTCTTGGGCTATCACAGAAACCTATTTATGTTCATGCTGAG
CAGCACACCATAGAGAAGAATCCAAATGATAGCCTTGACACCCTTGAAGCTATTCCTGA
TGCGGTTCCTGTTGTGTTGACTGAACCTCCAATTGAAGATCAATTGGGATGGCATACAC
TATGGCCAGAATCACATAAGCTTTATGGTCACGGGAATGAGCTGTTTGCTCTTTGCAGT
GATCATGAAGGGAAGCTTGTCGCTTCATCTTGTAAGGCCCAATCAGCAGCAGTTGCAG
AAATATGGCTGTGGGAAGTTGGTTCCTGGAAAGCAGTTGGTCGCTTGCAGTCGCATAG
TTTGACAGTTACACAAATGGAGTTCTCTCTTGATGACAAATTCCTGTTGGCAGTATCAA
GGGATCGCCAGTTCTCTATATTTTCAATTGACAAAACAGGCACTGATGGCACTAGTTAC

AAGCTGGTAGCAAAGCACGAGGCACACAAAAGAATCATATGGTCATGTTCCTGGAATC
CCCACGGCTATGAATTTGCAACAGGCTCAAGAGACAAGACGGTGAAGATCTGGACTGT
TGGAAAGGATTCATCAGTTAAGCTGCTCATGACTCTCCCACAGTTCAGCAGTAGCGTC
ACATCCCTATCTTGGGCTGGTCTTGATTCCAAGAAAAATAATGGGGTTCTTGCAGTTGG
AATGGAAAGCGGACTTATTGAATTGTGGAGTCTGTCTGTTAACAGAACCGATGATGGT
GTAGCAGCAAATGTACTTGCTACTCTTGTAGCACGGTTTGATCCATTGATGTGCCATGTT
TCTTCTGTGAGCCGTTTGGCGTGGAGAAAGCGCAAGAATAAAGATTGTACTAGCATAC
AGCTTGCCTCATGTGGAGCTGATCATTGTGTGAGAGTGTTCGAGGTAAAATGTTAA

>FvWD40-170

ATGAGCCAACAACCGTCTGTGATACTTGCGACGGCGAGCTATGATCACACCATTCGCTT
CTGGGAGGCCAAAGGTGGCCGCTGCTACCGCACCATCCAATACCCTGATTCACAAGTA
AACAGGCTCGAGATAACCCCGGATAAACGATTCCTGGCTGCAGCGGGCAATCCTCATAT
TAGGTTGTTTGATGTTAATTCAAACAGTCCTCAGCCGGTGATGAGCTATGATTCGCATAC
TAATAACGTTATGGCAGTGGGGTTTCAATGTGACGGGAACTGGATGTATTCAGGTTCCG
AGGATGGTACAGTAAAAATATGGGATTTGAGAGCTCCAGGTTGCCAAAGAGAATATGA
AAGCCGTGCAGCTGTTAACACTGTTGTGCTGCACCCGAATCAGACTGAACTAATATCTG
GGGACCAAAATGGCAACATCCGTGTTTGGGATTTGACAGCGAACTCTTGCAGTTGTGA
ATTGGTACCAGAGGTGGATACTGCTGTAAGATCACTAACAGTCATGTGGGATGGGAGC
TTAGTTGTTGCAGCAAATAATCATGGGACATGCTATGTTTGGCGCTTGTTGCGAGGGAC
ACAGACTATGACAAATTTTGAGCCACTTCATAAGCTTCAAGCACACAAGGGATATATCC
TCAAATGTCTACTTTCACCTGAGTTTTGTGAGCCCCACAGATATCTGGCCACTGCCTCT
TCTGACAACACTGTCAAGATATGGAATGTTGATGGTTTCACATTAGAAAAGACTCTTGA
AGGGCATCAACGGTGGGTCTGGGACTGTGTTTTCTCCGTAGATGGTGCCTATCTTATAA
CAGCTTCCTCTGATACAACAGCAAGGCTTTGGAACCTGTCCACCGGAGAAGATATCAG
AGTGTATCAGGGACATCACAAAGCAACCATTTGTTGTGCACTGCATGATGGAGCTGAA
CCTGGTCCACCTTGA
